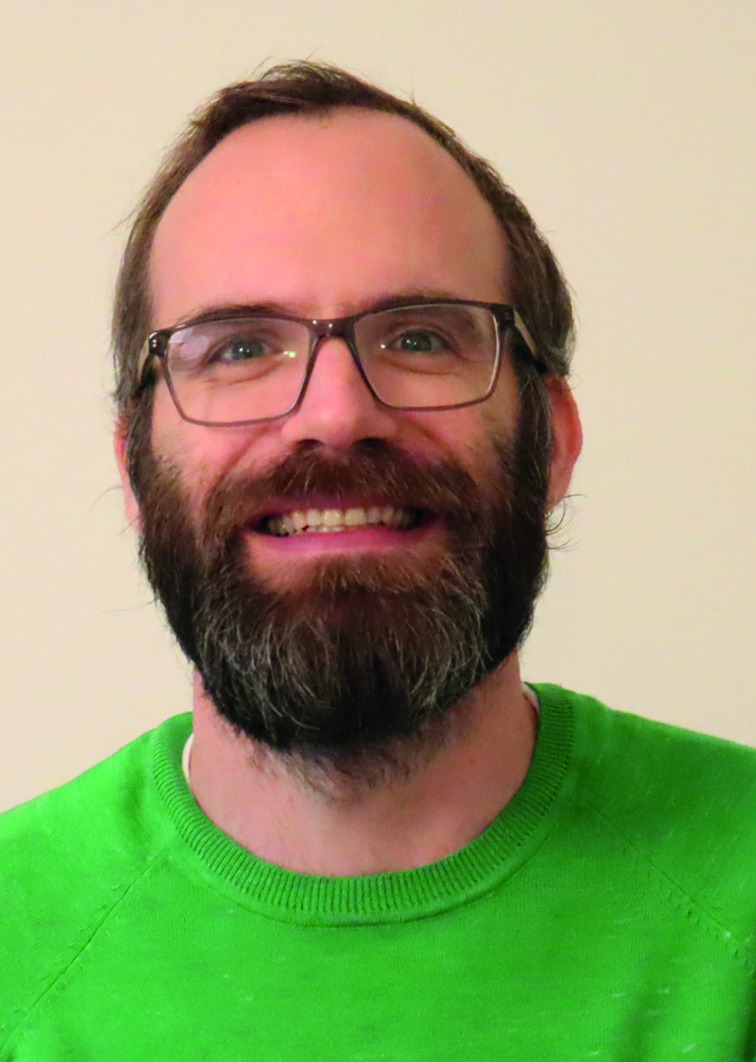# Maintaining the Integral Membrane Proteome: Revisiting the Functional Repertoire of Integral Membrane Proteases

**DOI:** 10.1002/cbic.202500048

**Published:** 2025-03-18

**Authors:** Hannah Fremlén, Björn M. Burmann

**Affiliations:** ^1^ Department of Chemistry and Molecular Biology Wallenberg Centre for Molecular and Translational Medicine University of Gothenburg 405 30 Göteborg Sweden; ^2^ Department of Chemistry and Molecular Biology Wallenberg Centre for Molecular and Translational Medicine Science for Life Laboratory Swedish NMR Centre University of Gothenburg 405 30 Göteborg Sweden

**Keywords:** Protein quality control, Integral membrane protein proteases, rhomboid proteases, AAA+ proteases, signal peptidases

## Abstract

Cells in all kingdoms of life employ dedicated protein quality control machineries for both their cytosolic and membrane proteome ensuring cellular functionality. These crucial systems consist besides a large variety of molecular chaperones, ensuring a proper fold and consequently function of the client's proteome, of several proteases to clean out damaged, unfunctional and potentially toxic proteins. One of the key features underlying the functional cycle of these quality control systems is the inherent flexibility of their bound clients which for a long time impaired detailed structural characterization, with advanced high‐resolution NMR spectroscopy in the last decade playing a key role contributing to the present understanding of their functional properties. Although these studies laid the foundation of the present knowledge of the mechanistic details of the maintenance of cytosolic proteins, the understanding of related systems employed for membrane associated as well as integral membrane proteins remains rather sparse to date. Herein, we review the crucial contributions of structural and dynamical biology approaches, possessing the power to resolve both structure and dynamics of such systems as well as enabling the elucidation of the functional repertoire of multimeric proteases involved in maintaining a functional membrane proteome.

## Graphical abstract

Regulated intramembrane proteolysis is crucial for functional proteome in all kingdoms of life. The task of cleaving transmembrane proteins within the unique environment of the lipid bilayer presents biochemical challenges that has been solved by integral membrane proteases (IMPRs). This review explores the structural and functional characteristics of IMPRs while also discussing future directions in this exciting field of science.

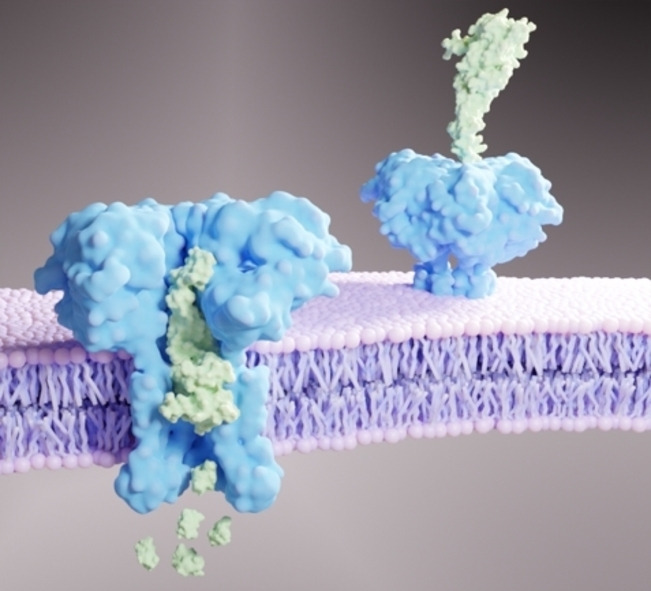

## 1 Introduction

Membrane proteins constitute approximately 20–30 % of the whole cellular proteome across all organisms and are fundamental components of cell membranes, participating in a range of vital biological processes.[^1^, ^2^, ^3^] α‐Helical integral membrane proteins (IMPs) span cellular membrane at least once, relying on sophisticated insertion mechanisms enabling them to function as transporters, receptors, and/or enzymes.^[4]^ Within this subset of cellular proteins, integral membrane proteases (IMPRs) play a particularly important role as they perform regulated intramembrane proteolysis, thus being a mandatory component in the protein quality control machinery and the cellular homeostasis in these sub‐cellular compartments.[^5^, ^6^, ^7^] Due to their integral membrane nature, IMPRs are exposed to a chemically challenging environment of both hydrophilic and hydrophobic nature. This feature makes their characterization inherently difficult, and as a result, their functional cycles and underlying mechanism less well‐understood in contrast to their soluble counterparts (e. g. reviewed in[^8^, ^9^, ^10^]).

On a functional level integral membrane proteases can be categorized into four main families: Rhomboid proteases (serine proteases), Presenilin and signal peptide peptidases II (SPase II) (aspartyl proteases), Site‐2 proteases (S2P metalloproteases), and the glutamyl proteases.[^5^, ^11^, ^12^] Each of the mentioned families have the inherent ability to proteolytically cleave a large variety of substrates, thereby critically contributing to a wide range of cellular roles.^[13]^ Research on IMPRs has largely centered around their structures and substrate recognition processes, with recent studies starting to reveal substrate‐induced conformational changes that facilitate cleavage, thus providing initial glimpses into their functional cycles. However, many aspects of substrate specificity and regulatory mechanisms remain to be elucidated to reveal their full functional repertoire. The present review aims to provide an in‐depth examination of the structural and functional roles of integral membrane proteases found in bacteria and mitochondria, with a specific focus on their contributions to protein quality control, ranging from protein translocation, maturation and finally targeted degradation, supported by the latest research developments in this vibrant scientific field.

## 2 The Bacterial Rhomboid Protease GlpG

*Escherichia coli* (*E. coli*) GlpG is an integral membrane protease residing in the bacterial inner membrane, belonging to the widespread rhomboid family of intramembrane serine proteases.[^14^, ^15^] Rhomboid proteases are found in all kingdoms of life contributing to a wide range of fundamental cellular processes such as signaling, regulation of mitochondrial homeostasis, and finally protein translocation.[^14^, ^15^] Bacterial rhomboids recognize and cleave single spanning integral membrane protein substrates within the lipid bilayer close to the periplasmic side. The proteolysis is conducted by a serine‐histidine catalytic dyad in the enzymes active site.^[16]^ Besides these rather general substrate preferences, the understanding of known substrates of bacterial rhomboids remains severely limited.^[17]^ So far, the only identified substrates are the twin‐arginine receptor TatA, the natural substrate of AarA, in *Providencia stuartii* as well as HybA and FdoH as GlpG substrates in *Shigella sonnei*.[^18^, ^19^, ^20^]

**Figure 1:**
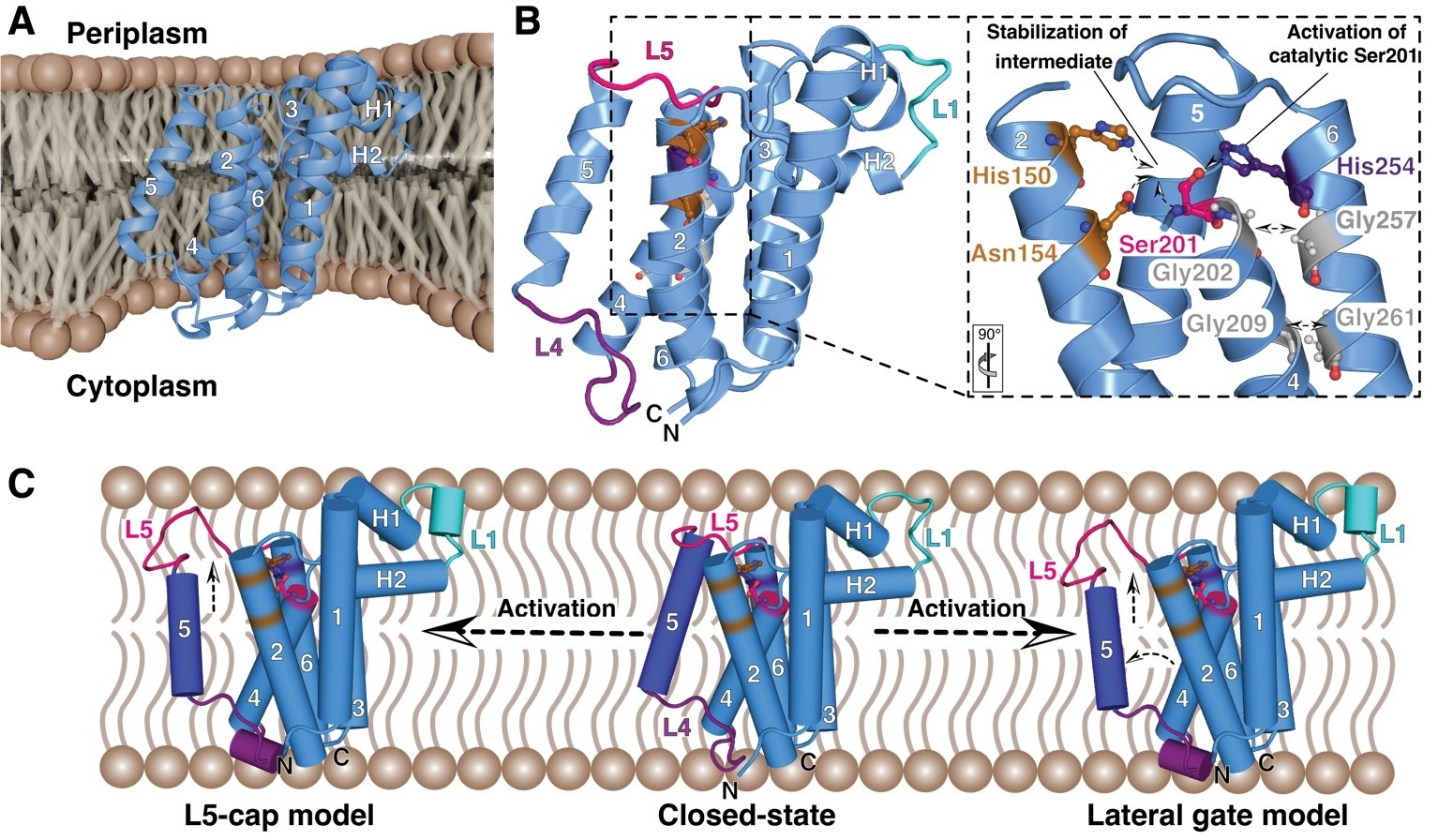
A) Crystal structure of the rhomboid protease GlpG (PDB‐ID: 2IC8) represented in the bacterial inner membrane. The membrane was created in Blender 4.3 (www.blender.org) and the GlpG structure was manually inserted to illustrate its incorporation in the membrane. This approach was used throughout the manuscript for visualizing the discussed proteins in their native lipid environment. B) View on the structural elements of GlpG. Termini and transmembrane helices are indicated. Discussed loops L1, L4, and L5 are labelled in cyan, purple, and pink, respectively, as well as the two membrane‐associated α‐helices H1 and H2. Zoom in onto the catalytic dyad composed of Ser201 (pink) and His254 (purple). His150 and Asn154 (orange) stabilize the intermediate state facilitating the cleavage of the peptide bond in the substrate. Important and evolutionary conserved glycines facilitating the close packing of α‐helices 4 and 6 are shown in grey. The respective orientation to panel A is indicated. C) Proposed activation mechanism of GlpG, either relying on the “lateral gate model” or the “L5‐cap model”. See main text for details. PDB‐IDs: 2IC8 (closed state), 2NRF (open state). Protein structures were visualized using either ChimeraX^[34]^ or PyMol 2.3.0.^[35]^

On a structural level, the GlpG core is composed of six transmembrane helices (denoted TM1–TM6), an overall topology shared by all known rhomboids. *E. coli* GlpG has an amino‐terminal cytoplasmic domain, lacking in some GlpG proteins found in archaea and thermophilic bacteria, with both termini facing the cytosolic side (Figure 1).[^21^, ^22^, ^23^] In between the membrane spanning helices short hydrophilic loops make the connections, with the sole exception of loop 1 (L1). L1 displays an extended loop region composed of several smaller α‐helices which is partially embedded within the membrane and positioned alongside the transmembrane helices, suggesting a crucial role in the GlpG functional cycle.[^16^, ^24^]

The architecture of the transmembrane core displays a remarkable feature, as in contrast to the other transmembrane segments TM4 does not span the complete membrane.^[16]^ This leads to a deeper position of TM4 within the lipid bilayer, with the catalytic serine (Ser201) positioned at the amino terminus of this helix 10 Å below the periplasmic surface (Figure 1). Another crucial structural feature is a central, cone‐shaped cavity composed of hydrophilic residues formed by adjacent transmembrane helices, which is filled with water molecules, needed for the hydrolysis of the peptide bonds, opening towards the periplasmic side (Figure 1B).^[16]^

Within this cavity lies also the catalytic histidine (His254) on TM6, which forms a strong hydrogen bond with the serine, together making up the catalytic dyad.^[16]^ To facilitate the interaction between the two catalytical residues in this environment, conserved glycines (Gly202 on TM4 and Gly257 and Gly261 on TM6) are present in the vicinity to Ser201 and His254. This arrangement enables close packing of the catalytic residue bearing helices TM4 and TM6.[^16^, ^21^] Crystal structures of GlpG in complex with inhibitors along with kinetic studies have shed light on the proteolytic mechanism of action. The catalytic serine (Ser201) is first activated by His254 for nucleophilic attack on the substrate carbonyl carbon forming a tetrahedral intermediate.

This intermediate is subsequently stabilized by the oxyanion hole comprised in the case of GlpG of unusual tripartite interactions with the sidechains of Asn154 and His150 as well as the backbone amide of Ser201 stabilizing *via* a water molecule the catalytic hydroxyl.[^16^, ^25^, ^26^, ^27^, ^28^] In the next step, the substrate peptide bond is cleaved, and the formation of an acyl enzyme occurs. A water molecule, proposedly activated by His254, then attacks the acyl enzyme complex and gives rise to the second tetrahedral intermediate followed by regeneration of the catalytic residues.[^26^, ^27^, ^29^, ^30^] Detailed insight into the catalytic cycle of GlpG was obtained by time‐resolved crystallography.[^25^, ^31^] By using varying times of incubation with different sized peptide inhibitors it was possible to visualize in total ten different states along the catalytic cycle of GlpG providing the foundation of the current understanding of its functional cycle.^[31]^

Despite this detailed structural insight, it has been an ongoing debate for almost two decades on how substrates gain access to the active site within the hydrophobic GlpG core.[^16^, ^28^, ^32^, ^33^] Two main mechanisms have been suggested, the “lateral gate” model and the “L5 cap” model (Figure 1C). The first model proposes that TM5 bends to form an entrance between TM2 and TM5, allowing substrates to enter the active site laterally.[^16^, ^32^] In contrast, the “L5 cap” model suggests a vertical substrate‐entry route where TM5 only moves slightly, and the main part of this opening is performed by L5 which lifts as a cap to expose the active site.[^28^, ^33^] Based on a recent study employing high‐resolution solid‐state NMR spectroscopy in combination with molecular dynamics simulations, complemented by functional assays, highlighted the direct relationship between lateral conformational changes of TM5 and protease activity, thus clearly in support of the lateral gate model.^[17]^ The obtained results showed increased proteolytic activity when mutations reducing the interaction between TM2 and TM5 were introduced. In line with this proposed functional principle, access to the catalytic site was almost entirely blocked when stable maleimide‐maleimide crosslinks between TM2 and TM5 were implemented.^[17]^ The “lateral gate” model was initially based on observations of an open and a closed state of GlpG in crystals.[^16^, ^23^] In the closed structure, L5 tightly seals the central cavity,^[16]^ whereas in the open conformation, the gating helix TM5 is rearranged and pulls away L5 upon proteolytic cleavage.^[23]^ One apparently common feature of both proposed mechanisms is the involvement of L5 for substrate entry, which is supported by the influence of mutations in this protein region modulating GlpG proteolytic activity .^[36]^

The existence of open and closed states was further elucidated by using high‐resolution magic angle spinning (HR‐MAS) solid‐state NMR spectroscopy where relaxation dispersion data confirms the structural interconversion between these two distinct states on a time scale of ∼40 μs.^[37]^ This NMR study revealed the origin of this movement to be the amino‐terminal part of TM5 in conjunction with the adjacent L4, suggesting a lateral gate opening.^[37]^

Besides these dynamical properties, the authors also observed a kink (Trp236) at the center of TM5 based on the carbon secondary chemical shifts, indicating the importance of this region in the lateral opening of GlpG.^[37]^ Remarkably, this observation agrees with earlier biochemical characterizations of the mutation of adjacent Leu234 to a helix‐breaking proline residue leading to increased proteolytic activity, suggested to be due to an extended lateral opening providing easier access to the catalytic site.^[32]^

Latest research focuses on the crucial question how GlpG interacts with the surrounding lipids of the membrane and how does this interplay influence its activity.[^38^, ^39^, ^40^] It was shown that GlpG is able to locally readjust the membrane, thinning a portion of the lipids that embrace the protein,^[38]^ a feature that also has been reported for a variety of membrane protein insertases.[^41^, ^42^, ^43^, ^44^, ^45^] It is believed that this local membrane thinning facilitates diffusion of GlpG[^38^, ^46^] by minimizing the hydrophobic mismatch, as the protease scans the membrane for substrates and potentially enhancing substrate interaction. A direct correlation between membrane thickness and proteolytic activity could be established for GlpG where an optimized hydrophobic thickness of the lipid bilayer resulted in the most efficient substrate proteolysis.^[39]^

The study of membrane proteins always faces the challenge of the protein being extracted from its natural environment, using for example detergent micelles, which can influence the structure, and the function compared to its native state where it interacts with the lipids of the membrane.^[47]^ To overcome this shortcoming, a recent paper by Sawczyc *et al*. exploited a method to examine weak protein‐lipid interactions for GlpG in a more native like environment without the use of detergents.^[40]^ The work was based on the previous development of synthetic polymers, such as styrene maleic acid (SMA) and diisobutylene maleic acid (DIBMA), which have the ability to solubilize a membrane protein together with its directly surrounding native lipids to form nanoparticles referred to as native nanodiscs (SMALPs/DIBMALPs).[^48^, ^49^, ^50^] Initial experiments using these solubilization tools were focused on GlpG to provide a more native lipidic environment, although they could not be used to also study GlpG in conjunction with native integral membrane proteins.[^51^, ^52^] Recently, it could also be shown that these compounds facilitate the lipid transfer from one nanoparticle to another, a property coined ‘collisional lipid mixing’, opening an avenue to characterize GlpG potentially with auxiliary proteins in these nanodiscs.[^48^, ^53^] Sawczyc *et al*. extended this initial usage of the nanoparticle property and showed that this transfer also occurs with protein‐containing SMALPs/DIBMALPs, thus paving the ground for studies at the atomic level of IMPRs together with their substrates in the future.^[40]^ As a first key result the authors could show that on the one hand GlpG protease activity is enhanced compared to detergent micelles and *en par* with the level observed in native bilayers, thus reflecting a more natural situation within these types of nanodiscs. Further, the authors succeeded in reconstituting a protease:substrate complex formed by GlpG and its substrate TatA, likely enabling future detailed structural and mechanistical investigations.^[40]^

## 3 The Bacterial S2P Peptidase RseP

RseP is an important IMPR found in *E. coli* belonging to the family of zinc dependent site‐2‐metalloproteases (S2P) with homologs found wide‐spread in bacteria, archaea and eukaryotes.[^54^, ^55^] The RseP protease is localized to the bacterial inner membrane and involved in the biogenesis and subsequent quality control of the bacterial envelope structure.^[56]^ RseP generally cleaves transmembrane substrates that span the membrane once with the amino‐terminal domain exposed to the cytoplasm and the carboxy‐terminal domain in the periplasm.[^57^, ^58^] In addition RseP plays a crucial role in the bacterial stress response pathway, as outlined in more detail below, and is involved in protein quality control of the cytoplasmic membrane proteome by degradation of fragmented signal peptides detached from secretory protein precursors during membrane translocation.[^57^, ^59^]

Initially, the topology of bacterial RseP was predicted to contain four TM segments while both amino‐ and carboxy‐termini are exposed to the periplasmic side.^[60]^ TMs 1–3 are highly conserved within the S2P family, whereas the last transmembrane helix (TM4) forms a less conserved single TM segment, creating a hydrophilic compartment adjacent to the catalytic site.[^54^, ^61^] For a long time, the only available crystal structure of a S2P group III member was of the archaeal homolog from *Methanocaldococcus jannaschii* with a median resolution of 3.2 Å.^[62]^ In contrast to the other classes, this protein lacks on the one hand auxiliary periplasmic domains and instead of the canonical site‐2 proteases harboring four transmembrane helices, it contains six of these.^[62]^ Based on crystal structures of an open and a closed state the authors were able to derive a possible gating mechanism, modulated by movements of helices TM5 and TM6 governing the accessibility of the membrane embedded catalytic site.^[62]^ Nevertheless, due to the uniqueness of these helices to group III S2P proteins no general conclusions about the mechanism could be drawn.[^62^, ^63^]

Recently, high‐resolution crystal structures of both *E. coli* RseP and its ortholog from *Kangiella koreensis (K. koreensis)* in complex with the peptide‐mimetic batimastat, a synthetic inhibitor of matrix metalloproteases,^[64]^ were reported.^[63]^ The structure confirms the predicted topology, of four TM helices, and provides more insight into the structural elements, as these group I S2P contain auxiliary PDZ domains lacking in group III members.^[55]^ The transmembrane segments are connected by loops where the L1‐loop situated between TM1 and TM2 displays a unique feature: L1 extends into the cytoplasm from TM1, enters the membrane to again extend into the cytoplasm before it connects with TM2.[^62^, ^63^] This loop forms a unique intramembrane β‐hairpin like structure forming a four stranded β‐sheet, termed MRE β‐loop (membrane‐reentrant β‐loop), creating a hydrophobic region between these two TMs.[^57^, ^63^] Furthermore, RseP constitutes two tandemly arranged PDZ domains (PDZ tandem) in the periplasm, PDZ−N on TM2 and PDZ−C on TM3.[^65^, ^66^] PDZ domains, named after the first proteins PSD‐95, Dlg1, and ZO‐1, in which this type of protein domains was observed, are found in a variety of different types of proteins where these domains being commonly involved in substrate recognition.^[67]^ For cytoplasmic and periplasmic proteases of the high‐temperature requirement A (HtrA) family of serine proteases such as DegS, DegP, and human HtrA2, the PDZ domains also modulate the access of substrates to the catalytic sites and play a key role in the activation steps of the proteolytic function of HtrA‐proteins.[^68^, ^69^, ^70^, ^71^, ^72^]

**Figure 2:**
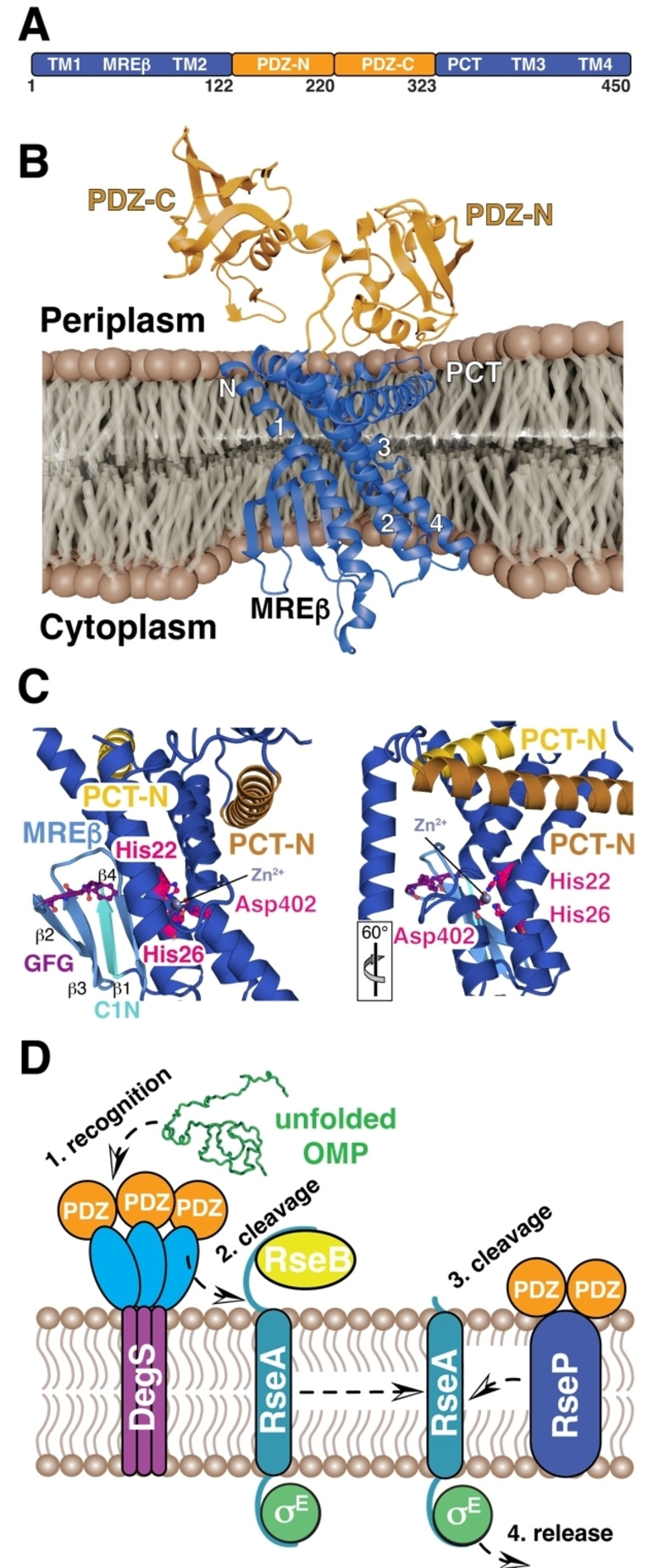
A) Schematic representation of the RseP structural elements with the integral membrane parts shown in dark‐blue and the soluble domains in orange. TM indicates the different transmembrane helices (1–4), MREβ the four stranded membrane‐reentrant β‐loop structure, PCT the PDZ carboxy‐terminal region and finally the two soluble PDZ domains termed PDZ−N and PDZ−C. B) Crystal structure of full‐length *E. coli* RseP (PDB‐ID: 7W6X) represented in the bacterial inner membrane. Structural elements are indicated. C) Two close‐up views onto the catalytic site. Catalytic residues His22, His26 and Asp 402 are highlighted in pink. Regulatory elements such as the MREβ (blue), GFG motif (purple), C1 N (cyan), PCT−N (orange), and PCT−C (yellow) are labeled. See main text for details. The orientation of the second view relative to the first one is indicated. D) Extracytoplasmic stress leads to the accumulation of unfolded or denatured outer membrane proteins (OMP, green), which in the first step is recognized by the PDZ‐domains (orange) of the membrane associated HTRA‐protease DegS (blue, purple). This activates DegS proteolytic function leading to the carboxy‐terminal cleavage of the anti‐sigma factor RseA (cyan, site‐1 cleavage). Subsequently, RseP (dark‐blue) proteolytically cleaves the resulting RseA within the membrane (site‐2 cleavage) leading to the release and activation of the σ^E^ (green) stress response pathway. RsePs two PDZ‐domains are supposed to sterically hinder cleavage of unprocessed RseA.

Within RseP, the PDZ tandem is localized at the center of the periplasmic region and forms a pocket towards the TM segment containing the active center.^[61]^ This tandem pocket is proposed to act as a size‐exclusion filter to tightly regulate substrate entry to the active site (Figure 2A, B).[^65^, ^73^] With this approach, substrate discrimination is thus solely based on the size of the substrate's periplasmic domain rather than a specific degron motif.^[65]^ The active site of RseP is embedded within the hydrophobic lipid bilayer and exhibit the conserved zinc binding motif H^[22]^ ExxH^[26]^ on TM1 together with the LD^402^G sequence on TM3 (Figure 2C).[^63^, ^74^] Since the catalytic zinc ions are dependent on access to water molecules for efficient hydrolysis, RseP must form a hydrophilic region to permit entry of water into the active site. This hydrophilic environment within the membrane is lined by several basic residues on the cytoplasmic side of the MRE β‐loop which exclude lipid molecules around the loop on the membrane to allow the passage of water molecules.^[63]^

The region of the first cytoplasmic loop (C1) that is located amino‐terminally adjacent to the MRE β‐loop, named C1 N, has also been investigated in relation to substrate binding and cleavage (Figure 2C). C1 N is partially inserted into the membrane domain with a β‐hairpin‐like structure and contains a conserved G^43^FG motif, which has a crucial role in the proteolytic activity of RseP. A previous study conducted systematic mutagenesis of the C1 N region as well as co‐immunoprecipitation and *in vivo* crosslinking experiments with results suggesting that the GFG motif of C1 N binds to the substrate. Substrate binding by the C1 N region seems to be required for efficient proteolysis and results indicate that substrate binding occurs both at the GFG motif in C1 N and at the binding site sequence in the MRE β‐loop. The exact mechanism of how the substrate binds, i. e., if the substrate binds the two binding sites simultaneously or first to the C1 N region to then be transferred to the MRE β‐loop remains elusive.^[74]^ Serendipity lately helped to shed more detailed light into these crucial questions as the *Aa*RseP (*Aquifex aeolicus* RseP) structure could be solved in the presence of an endogenous substrate from the expression host.^[75]^ The obtained structural data revealed an extended conformation of the substrate, TM9 of the *Ec*CyoE integral membrane protein, presumably achieved through substrate threading towards the RseP active site, in good agreement with previously reported experimental data.[^63^, ^73^, ^75^]

RseP proteolysis occurs within the membrane after the substrates have been subjected to initial extracytoplasmic cleavage by site‐1 proteases.^[63]^ RseP is a key component in the critical process of regulating the σ^E^–cascade pathway in stress response to misfolded proteins within the cellular envelope to ensure cell viability (Figure 2D).[^76^, ^77^, ^78^, ^79^] σ^E^ remains inactive during normal growth conditions by interaction with the membrane protein RseA, which is a so‐called anti‐sigma‐factor.[^80^, ^81^] Upon envelope stress response, another membrane associated protease, DegS, is activated and introduces site‐1 cleavage into the RseA periplasmic region.[^82^, ^83^, ^84^] The partially cleaved RseA is subsequently introduced to RseP for site‐2 cleavage at the cytoplasmic site and is subsequently degraded.^[57]^ A range of *in vivo* and *in vitro* studies show that RseP can cleave a limited number of other single TM sequences.[^58^, ^85^, ^86^] Alike the previously described GlpG protease, so far there has been no observations of a common recognition motif within the TM segments of the different RseP substrates and the previous examples share no apparent sequence similarity to each other or to the canonical substrate RseA.^[57]^ However, like other IMPRs discussed herein, the stability of the helical TM domains and presence of helix‐destabilizing residues interacting with the MRE β‐loop seem to be a pre‐condition for susceptibility to RseP cleavage.[^57^, ^58^]

## 4 Bacterial Signal Peptide Peptidases

**Figure 3:**
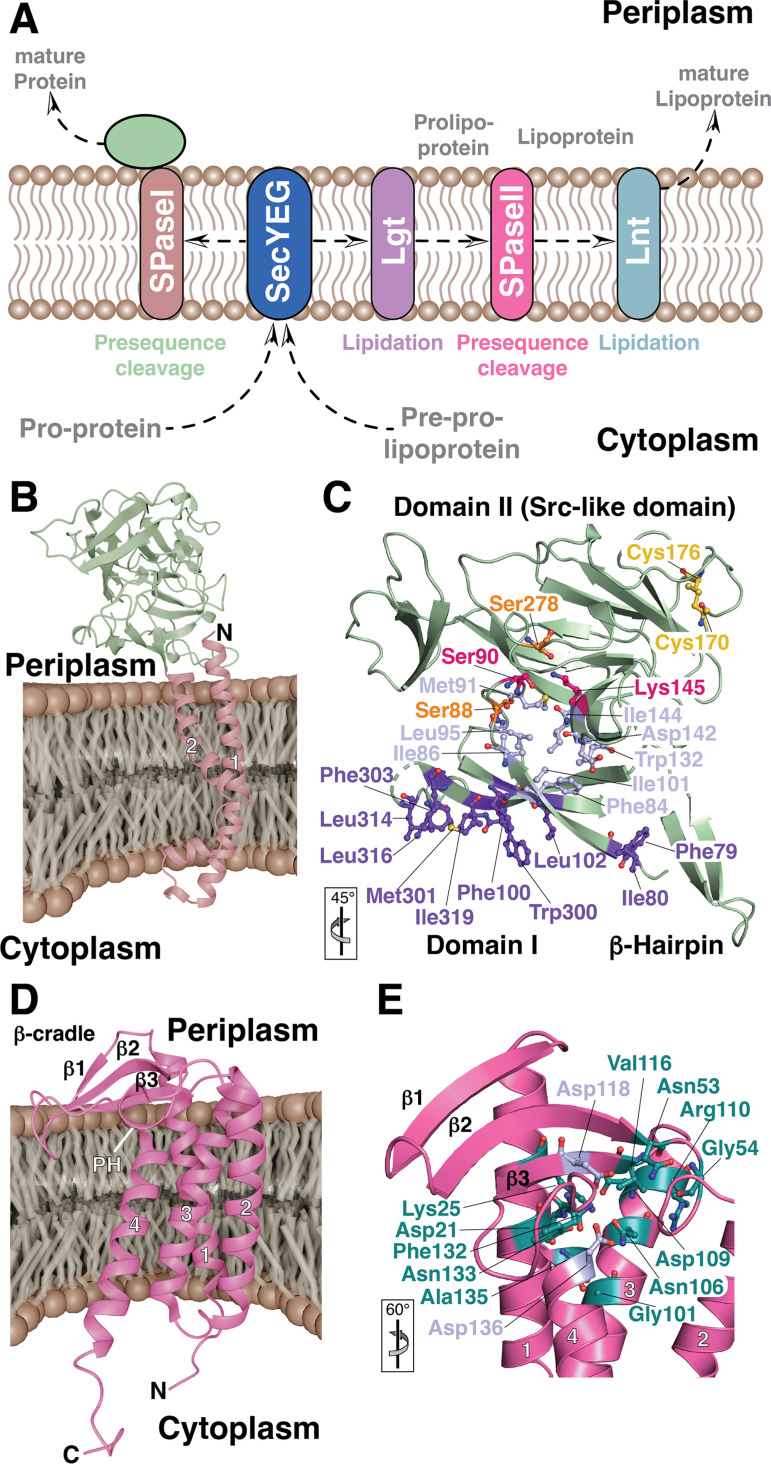
A) Scheme of transport and subsequent processing of proteins passing the bacterial inner membrane. The vast majority of the preproteins are translocated *via* the SecYEG (blue) channel and processed by the SPaseI (red/green) to cleave the signal sequence. The mature proteins reside either in the periplasm or are transported to the bacterial outer membrane (left side). The lipoprotein posttranslational processing pathway in Gram‐negative bacteria is depicted on the right‐hand side. After translocation by SecYEG the pre‐prolipoteins are modified with a diacylglycerol by Lgt (purple). Subsequently, the SPaseII (LspA, magenta) removes the signal peptide, and in a last step Lnt (cyan) adds an N‐acetylate to the amino‐terminal cysteine. The mature lipoprotein is shuttled to the outer membrane by the LOL transportase. B) Alphafold3^[98]^ generated model of the full‐length *E. coli* SPaseI embedded into the bacterial inner membrane. C) Zoom in onto the crystal structure of the periplasmic domain of SpaseI (PDB‐ID: 1B12). The catalytic residues Ser88 and Lys278 (magenta), residues stabilizing the transition state, Ser90 and Ser278 (orange), as well as the substrate binding groove (light blue) are highlighted. In addition, hydrophobic residues attaching the domain to the inner membrane are show in purple and the characteristic disulfide bridge between Cys179 and Cys176 (yellow). D) Crystal structure of LspA from *Staphylococcus aureus* (PDB‐ID: 6RYP) represented in the bacterial inner membrane. β‐Cradle and PH (periplasmic helix) structural elements are labeled. E) Close‐up view on the catalytic site formed by Asp118 and Asp136, respectively (blue). Conserved residues are shown in cyan.

Bacterial proteins destined for transport across the bacterial inner membrane contain specific target sequences enabling the SecYEG machinery, embedded within the membrane, to translocate these proteins into the periplasm.[^87^, ^88^, ^89^, ^90^] After translocation, these target sequences, termed signal peptides, are cleaved of by dedicated signal peptidases.^[91]^ Within bacteria two parallel systems exist (Figure 3A): on the one hand the serine protease SPaseI which cleaves off the signal peptides of most translocated proteins^[91]^ and on the other the aspartyl protease SPaseII (LspA), which specifically cleaves the signal peptides of ~100 lipoproteins transported.[^92^, ^93^]

SPaseI is a classical serine protease relying on a serine‐lysine dyad.^[94]^ The protease is anchored to the inner membrane by two helices, whereas the catalytic center resides in the periplasmic domain (Figure 3B).^[94]^ The structure determination by X‐ray crystallography of the periplasmic domain of SPaseI revealed a unique structure:^[95]^ several coiled β‐sheets in addition to an SH3 barrel domain,[^95^, ^96^] a classical protein domain fold commonly associated with intramolecular associations important for protein regulation.^[97]^ The catalytic center is embedded within domain I, formed by the Ser90 and Lys145 dyad (Figure 3C).^[95]^ Substrate specificity is achieved by an extended hydrophobic pocket engaging with the characteristic alanine residues of the pre‐sequences (Figure 3C).^[99]^ Detailed analysis of this binding pocket revealed that, in particular, Ile86 and Ile144 are crucial for specificity and cleavage site fidelity.[^100^, ^101^] The close association to the inner membrane of the periplasmic SPaseI domain is achieved by an extended hydrophobic surface lining the outside of Domain I, potentially crucial for the exact positioning of the catalytic center in the vicinity of the membrane. This observation was corroborated by earlier biophysical investigations indicating that the catalytic domain might need to be partially embedded in the membrane facilitated by phosphatidylethanolamine, the most abundant lipid in the inner membrane of *E. coli*.[^102^, ^103^, ^104^]

For characterizing the interaction between SPaseI and a signal peptide, NMR spectroscopy was employed.[^105^, ^106^] These studies confirmed the importance of helix‐breaking residues within the signal peptide to ensure engagement with the catalytic site, reminiscent of the structure reported for a signal peptide bound to the SecYEG adaptor SecA.[^99^, ^106^, ^107^]

Besides the general signal peptidase SPaseI bacteria contain a second signal peptidase, the unique aspartyl SPaseII (LspA).^[87]^ This peptidase acts on lipoproteins together with two other modifying enzymes: the phosphatidylglycerol/prolipoprotein diacylglyceryl transferase (Lgt), adding the lipid to the prelipoprotein, and subsequently getting amino‐acylated by phospholipid/apolipoprotein transacylase (Lnt) generating the mature triacylated lipoprotein (Figure 3A).[^87^, ^108^, ^109^] LspA is on the sequence level a unique signal peptidase that is found exclusively in bacteria, playing an essential role in the posttranslational processing of lipoproteins of gram‐negative bacteria, thus making it a potentially highly effective target for antibiotics.[^91^, ^110^] LpsA is a small protein of around 170 residues. Only recently, the structures of the *Pseudomonas aeruginosa (P. aeruginosa)* and *Staphylococcus aureus (S. aureus)* were solved by crystallography.[^110^, ^111^] To obtain the protein structures, the researcher's had to resort to crystallization by the meso (lipidic cubic phase) method[^112^, ^113^] in the presence of an antibiotic.[^110^, ^111^] By this approach, membrane proteins are embedded in a lipidic cubic phase enabling the integral membrane protein to interact directly with lipid molecules while representing a more similar environment to the inner membrane in comparison to detergent micelles.^[114]^ Crystallization was only possible with the LCP technique and in the presence of an antibiotic acting as a non‐cleavable peptide analogue,[^110^, ^111^] pointing to inherent flexibility and potential functional dynamics of LpsA itself in the substrate‐free state.[^110^, ^111^]

The obtained crystal structures of LspA from the two bacterial species in the presence of the antibiotic globomycin, a cyclic lipodepsipeptide originally isolated from *Streptomyces sp*.,^[115]^ revealed a similar state:[^110^, ^111^] the enzyme contains four transmembrane helices with the catalytic dyad aspartates (*Sa*LspA: Asp118, Asp136; *Pa*LspA: Asp134, Asp143) pointing to the outer leaflet of the inner membrane (Figure 3 D). Protruding into the periplasmic space and oriented on the site of the transmembrane helices, three β‐strands form a hemi cylindrically β‐cradle that is connected to the transmembrane part with a highly conserved periplasmic helix (PH), blocking access to the active site.[^110^, ^111^] These two structural features are expected to enable the binding to residues carboxy‐terminally to the cleavage site of lipoprotein substrates.^[111]^ Besides these structural features, both structures show an extended patch of fourteen highly conserved residues lining the active site as well as the signal peptide binding pocket (Figure 3 E).^[110]^ This high degree of conservation points to an importance of this region and indicates that antibiotic resistance mutations arising within this patch would impair the essential function of LspA, highlighting the potential of LspA as an antibiotic target.^[116]^ Even though the structures were obtained without a native substrate, the identification of monoolein lipids originating from the cubic meso phase suggest a stabilization of the native acyl chains of lipoprotein substrates.^[111]^ Interestingly, the *Sa*LspA structure in the presence of another antibiotic, myxovirescin, a macrocyclic secondary metabolite originally isolated from *Myxococcus verescens*,^[117]^ showed subtle structural differences besides a similar mode of binding at the catalytic dyad.^[111]^ Overall, this natural compound interacted with a different site in the substrate binding pocket leading to differences in the PH suggesting inherent flexibility potentially needed to interact with a range of native substrate lipoproteins.^[111]^ Comparing the two available *S. aureus* structures indicates the ability of LspA to adapt to different substrates and undergoing open‐to‐close transitions modulated by the PH.^[116]^ These conformational dynamics were subsequently probed by molecular dynamics calculations, which in combination with electron paramagnetic resonance studies (EPR), revealing large rearrangements in the cradle region on the nanosecond timescale, enabling access to a variety of substrates to the active site.^[116]^

Besides the crystal studies described above relying on the LCP methodology, pioneering studies by solution NMR spectroscopy highlighted the usage of protein nanodisc‐derived isotropic bicelles.[^118^, ^119^] In agreement with prior structural studies, initial test with detergent micelles resulted in unstable samples leading to poor quality NMR spectra, which could be improved by the usage of protein nanodiscs.^[118]^ In this approach, membrane protein in detergents are transferred to lipid:MSP protein mixture, where the MSP protein provides a rim around the lipid molecules enabling the incorporation of integral membrane proteins.[^120^, ^121^, ^122^] Even though this approach yielded better NMR‐samples, the overall quality proved not to be sufficient without the subsequent addition of detergent molecules leading to isotropic bicelles.^[118]^ Further evidence of crucial protein lipid interactions stabilizing LspA:lipid contacts could be derived by the characterization of several protein:lipid NOEs (Nuclear Overhauser effect) reporting on direct and specific proton:proton contacts between protein and lipid.^[118]^

## 5 The Bacterial AAA+ Protease FtsH

**Figure 4:**
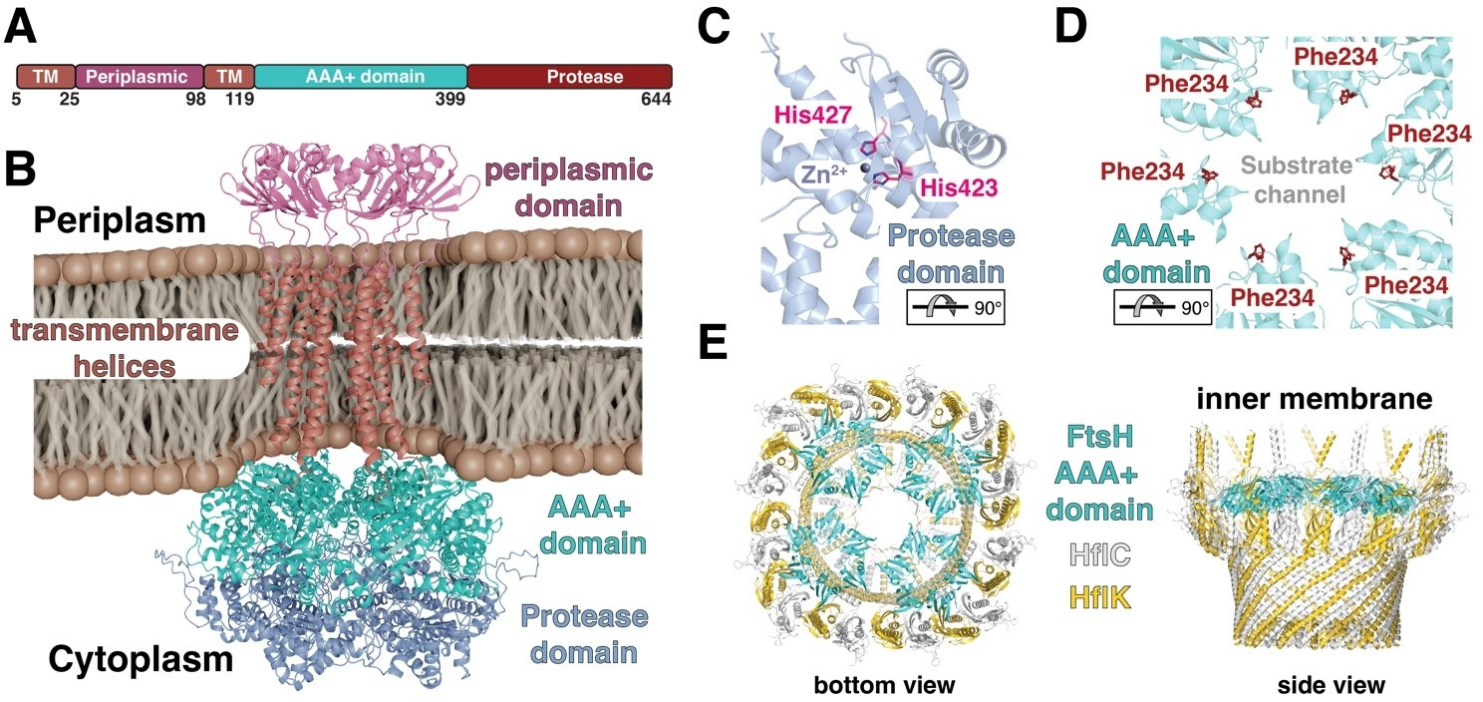
A) Schematic representation of the structural elements of the FtsH monomer indicating the two transmembrane helices (TM1, TM2) in brown, the periplasmic domain in purple, AAA+ domain in cyan and the protease domain in dark‐red. B) Alphafold3^[98]^ generated model of the mature FtsH hexamer embedded into the bacterial inner membrane. C, D) Crystal structure of the cytoplasmic domain of *T. maritima* FtsH (PDB ID: 3KDS). Focus on the catalytic site of one protomer (C) formed by His423 and His427 (pink) coordinating the catalytic zinc ion. Close‐up view (D) onto the substrate channel within the AAA+ domain lined up by the crucial Phe234 (red). The rotations of the domains relative to panel A are indicated E) Medium resolution (3.4 Å) Cryo‐EM structure (PDB‐ID: 7VHP) complex of the symmetric Ftsh‐HflC‐HflK complex from *E. coli*.

The metalloprotease FtsH, which is localized within the inner membrane of *E. coli* and the only essential AAA+ (ATPases associated with various cellular activities) protease found in bacteria, markedly differs from the other integral membrane proteases discussed thus far:[^8^, ^123^] FtsH possesses two functional domains in the cytoplasm, an ATPase domain, required for protein unfolding and translocation, alongside its proteolytic domain, with both domains being connected to a small periplasmic domain by transmembrane helices (Figure 4). AAA+ proteases use energy in the form of ATP to initially unfold and subsequently degrade target proteins, which occurs in the case of FtsH outside the lipid bilayer.[^8^, ^123^, ^124^, ^125^] Proteolysis by FtsH requires the presence of zinc at the active site, and its native substrates include both soluble and membrane‐bound proteins.^[126]^ FtsH homologs are found across all species, in eukaryotic cells, playing also important quality control roles within mitochondria as well as chloroplasts.[^127^, ^128^]

Structurally, FtsH assembles into a homohexamer with a symmetric ring‐like structure.[^129^, ^130^, ^131^] Each monomer contains a large carboxy‐terminal cytoplasmic region constituting approximately 520 amino acids, two membrane spanning helices (TM) and a periplasmic amino‐terminal region of approximately 75 residues, which is located between the two TMs (Figure 4A). The amino‐terminus of the cytoplasmic region is connected to the TMs *vi*a a short glycine‐rich linker of approximately 20 residues.^[132]^ The carboxy‐terminal region comprises of both the AAA+ domain and the proteolytic domain, which are connected by a 12 residue long flexible linker.^[129]^ The AAA+ domain is responsible for unfolding and translocation of substrates into the proteolytic chamber of the protease domain, where degradation occurs. AAA+ proteases usually constitute several highly conserved characteristic sequence motifs which also can be found in FtsH. These include the Walker A and Walker B motifs, which bind and hydrolyze nucleotides, and the second region of homology (SRH) encompassing the so‐called “arginine finger” which is crucial for oligomerization and ATP hydrolysis. The protease domain itself contains a characteristic zinc binding motif, HEXXH (X represents any amino acid), where the two histidines in conjunction with an adjacent glutamic acid are responsible for co‐ordinating the catalytic Zn^2+^ ion (Figure 4C).^[133]^

Structural studies of FtsH homologs were initially restricted to using isolated constructs of the cytosolic region[^129^, ^134^, ^135^, ^136^, ^137^] and the periplasmic region,^[138]^ providing only limited insight into the underlying functional mechanism of FtsH. In recent years, cryo‐EM and negative stain electron microscopy structures of the full‐length FtsH of *Escherichia coli (E. coli)*,^[132]^ *Aquifex aeolicus (A. Aeolicus)*,[^139^, ^140^] and *Thermotoga maritima (T. maritima)* ^[140]^ have been reported, providing fresh insight into the structural properties of the full‐length protein. The different structures of the cytosolic region all show a stably formed hexamer of the protease domain, which consists of mainly α‐helical secondary structure elements supplemented with a short segment of β‐strands.[^129^, ^134^, ^135^, ^136^, ^137^] This symmetric hexameric ring forms a central cavity composed of the conserved residues MFVG, where the aromatic phenylalanine has been reported to play a crucial role by facilitating the substrate translocation into the chamber (Figure 4D),^[133]^ representing a typical aromatic staircase found in protein unfoldases in general.[^8^, ^141^, ^142^, ^143^] The AAA+ domain of FtsH is structurally also composed of mainly α‐helices complemented with five parallel β‐strands.[^129^, ^134^, ^135^, ^136^, ^137^] However, in contrast to the protease domain, the hexameric ring of the AAA+ domain does not seem to present a perfect sixfold symmetry but instead different oligomeric states in the obtained crystal structures were observed: AAA+ ring in an ADP bound state, has been shown in *T. maritima* to possess a twofold symmetry,^[134]^ in *A. aeolicus* as either a threefold^[129]^ or as a twofold,^[136]^ suggesting larger structural heterogeneity within this domain compared to the protease domain. These conformational differences within the AAA+ domain can likely be attributed to the large structural rearrangements underlying ATP hydrolysis, a feature observed also for related soluble AAA+ unfoldases, facilitating the threading of the unfolded substrate thorough the central pore of the respective unfoldase domains.[^124^, ^125^]

Unlike other integral membrane proteases described above, FtsH either interacts with soluble substrates or extracts its target membrane proteins from the membrane and subsequently degrades them in an ATP dependent manner.^[126]^ The substrates are recognized and initially bound by the AAA+ domain which unfolds and subsequently translocates them into the proteolytic chamber under ATP consumption.^[126]^ This process is dependent on ATP and driven by the conformational changes induced by ATP hydrolysis.^[133]^ Proteolysis is thereafter performed by the protease domain and the substrates are degraded into small peptide fragments which are released into the cytoplasm.^[127]^

Since the active sites are localized in the center of the ring‐shaped domains, substrates can only enter through a narrow pore consisting of several aromatic amino acids, which are important for both substrate binding and subsequent translocation.^[144]^ The architecture of the entry pore is believed to act as a filter to allow only passage of unstable and partially unfolded substrates.^[130]^ Thus, ATP hydrolysis was initially believed to be mainly used for translocation of unfolded substrates into the proteolytic chamber using soluble model substrates bearing unfolded degradation tags at the protein termini.[^127^, ^145^] The unfoldase capability of the ATPase domain itself was considered to be relatively weak, suggesting to play a role in FtsH substrate discrimination based on target protein thermodynamic stability.[^127^, ^145^] However, more recent studies on the proteolytic degradation of the integral membrane protease GlpG, show an energetically highly effective process of membrane dislocation, unfolding and degradation for individual membrane helices,[^146^, ^147^] potentially guided by polar residues.^[148]^ Subsequently, studies on the model substrate dihydrofolate reductase (DHFR) revealed that FtsH does not require unfolded terminal tag‐regions for substrate recognition, but also in soluble protein substrates seems to recognize internal partially stable helical sequences.^[149]^

On a cellular level, FtsH plays a key functional role in bacterial protein quality control as it degrades misfolded and aggregated proteins while also performing regulated proteolysis under specific cellular conditions. Even though FtsH is an essential protease, very little is known about its complete substrate repertoire and the respective substrate‐recognition.^[126]^ FtsH can target both cytosolic substrates and membrane bound substrates, making FtsH a unique protease considering that most IMPRs target other membrane proteins solely.^[126]^ The vital function of FtsH in *E. coli* is to degrade the cytosolic enzyme LpxC which is involved in lipopolysaccharide (LPS) biosynthesis.[^150^, ^151^] LPS, together with phospholipids, acts as a permeability barrier in the outer leaflet of the outer membrane and impaired LPS concentrations can influence the integrity of this barrier and are thus directly lethal to the cell. Therefore, strict regulation of LPS and phospholipids relative concentration is required,[^152^, ^153^] as the LPS directly interacts with integral outer membrane proteins (OMPs) contributing to the mechanical stability of the bacterial cell.^[154]^ Another important cellular function of FtsH is the proteolysis of the heat shock σ factor (σ^32^), which regulates the transcription of heat shock proteins as a response to cellular stress. The activity of σ^32^ is modulated by the DnaK and GroEL/ES chaperones which partially unfold σ^32^ for subsequent FtsH‐dependent degradation and thus shutting off transcription of the stress response.[^155^, ^156^]

FtsH interacts with the single spanning membrane proteins HflK and HflC forming extremely large complexes within the inner membrane extending into the cytoplasm.[^157^, ^158^] The HflKC complex is thought to regulate the proteolytic activity of FtsH.[^132^, ^157^] Recently, two structures solved by cryo‐EM of the FtsH‐HflKC complex were published and showed a symmetric overall structure resembling a bell‐shaped cage, formed by 12 copies of HflK and HflC each, in the periplasmic space (Figure 4E). Four FtsH homohexamers are located inside this cage with the periplasmic domain and transmembrane segments buried inside and with the cytoplasmic domains at the bottom of the cage.[^132^, ^159^] The FtsH‐HflKC structure together with mutagenesis and activity assays could identify interactions between the β2–β3‐turn loop from two adjacent periplasmic domains of FtsH (residues Lys61, Asp62 and Ser63) and two HflK subunits, enabling assembly of the protein complex. The recently solved structures of the complex also provide more insight into how FtsH is regulated by HflKC. The architecture of the complex reveals how the membrane as well as the periplasmic domain of FtsH are covered by HflKC, thus prevented from degrading functional membrane proteins.[^132^, ^159^] This model suggests that HflKC functions as an adaptor that binds, then presents substrates to FtsH and might negatively regulate proteolysis of membrane proteins.[^132^, ^159^] However, latest findings using extraction of non‐overexpressed complexes, indicate that there might be also asymmetric FtsH‐HflC‐HflC complexes potentially illustrating the repertoire of FtsH modulation.^[160]^ Based on the cryo‐EM structures of these asymmetric complexes build up from 24 HflK/C subunits together with one or two FtsH hexamers revealed an open ring structure enabling direct access to the FtsH protease.^[160]^ These asymmetric assemblies might represent most of the cellular FtsH‐HflC‐HflC complexes as measurements of cellular protein stoichiometries indicate an average of ~2.3 FtsH hexamers for every 24 HflK/C subunits under a range of growth conditions.^[161]^

Even though interprotomer regulation and underlying dynamical changes upon ATP consumption of the FtsH protease remain largely unknown, recently, the potential of forming larger oligomeric species was reported for the isolated cytoplasmic domain.^[162]^ The formation of FtsH nanotubes in the presence of saturating amounts of ATP, partially resembles the formation of microtubili, even though the FtsH nanotubes lack efficient ATPase activity.^[162]^ The observation of large oligomeric assemblies for storage or hibernation function is potentially an inherent property of the AAA+ module as similar behavior has been reported for different AAA+ proteins residing in the cytoplasm: multimerization of DnaA enables replisome assembly,^[163]^ eukaryotic TorsinA involved in nuclear pore complex maturation,[^164^, ^165^] and the transcription termination factor Rho polymerizes under stress conditions as an additional layer of gene regulation.[^166^, ^167^]

## 6 Mitochondrial AAA+ Proteases

Bacterial FtsH has two homologs residing in the mitochondrial inner membrane (IM) of eucaryotic cells: the matrix AAA (*m*‐AAA) and the intermembrane AAA (*i*‐AAA) proteases.^[9]^ As their names suggest, these two AAA+ proteases display their proteolytic domains on opposite faces of the mitochondrial inner membrane.[^9^, ^10^, ^168^] With membrane‐spanning α‐helices anchoring them to the membrane, the *m*‐AAA protease has its domains exposed to the mitochondrial matrix whilst the *i*‐AAA protease exerts its catalytic activity in the intermembrane space (IMS).[^169^, ^170^] Like bacterial FtsH, both proteases contain one AAA+ domain for transportation and potentially unfolding of substrates to the proteolytic domain which harbors the other functional domain facilitating the zinc dependent degradation of its substrates.^[171]^ These two AAA+ proteases play a crucial role in the mitochondrial protein quality control system as they degrade misfolded or unassembled proteins on both sides of the inner membrane maintaining a functional mitoproteome.^[10]^ Whereas the *m*‐AAA protease removes both inner membrane as well as matrix proteins, the substrates of *i*‐AAA are residing in the intermembrane space as well as in both the inner and outer mitochondrial membrane.^[168]^

Structurally, both AAA proteases form hexamers with the functional domains forming ring shaped structures. Mammalian *i*‐AAA is formed by YME1L in human and by Yme1 in yeast, for both organisms, the structure is homohexameric while *m*‐AAA can create homo or heteromeric structures depending on species and location.^[10]^ Human *m*‐AAA constitutes of either AFG3L2 as a homohexamer or as a heterohexamer of both AFG3L2 and paraplegin while in yeast, the Yta10 and Yta12 subunits, respectively, forms the catalytically active hexamer.[^10^, ^168^, ^172^]

The crucial importance of these mitochondrial proteins is reflected by their role in human diseases; mutations in paraplegin can lead to a severe neurodegenerative disorder known as spastic paraplegia,^[173]^ mutations in AFG3L2 can lead to different types of ataxia, inherited brain disorders affecting physical movements,[^174^, ^175^] mutations in YME1L have been associated with mitochondrial dysfunction leading to severe intellectual disability, muscular impairments and optic nerve atrophy.[^176^, ^177^]

### 6.1 Mitochondrial i‐AAA Proteases

**Figure 5:**
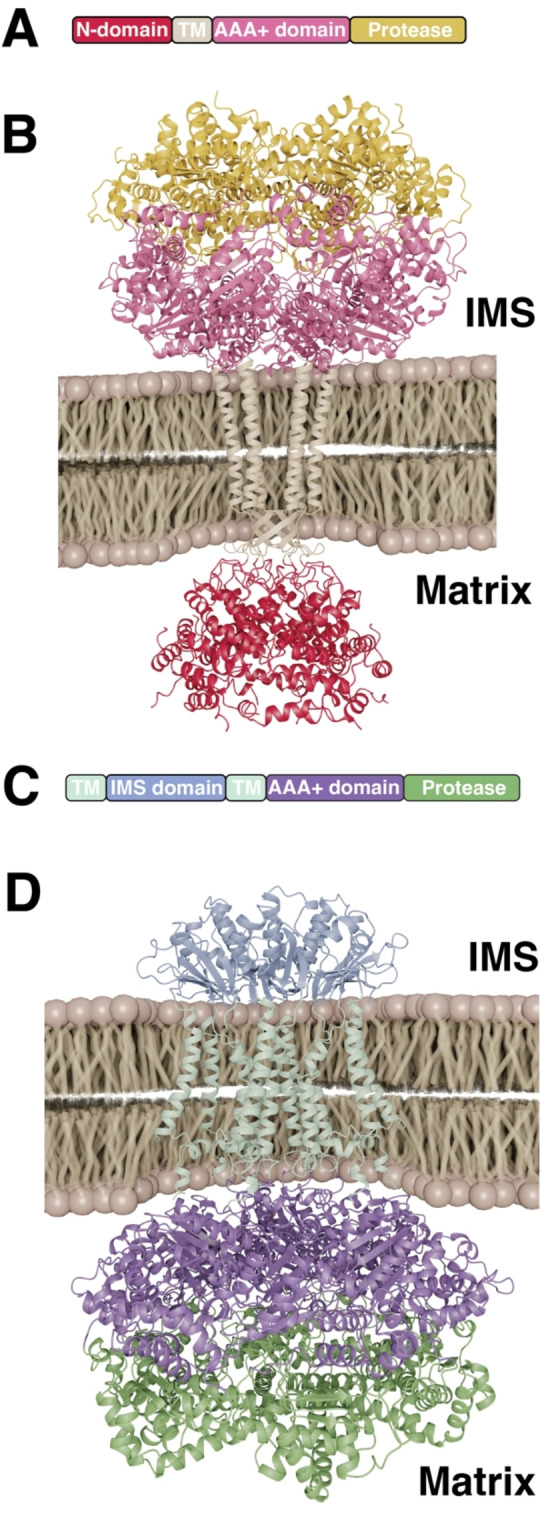
A) Schematic representation of the structural elements of the *i*‐AAA monomer indicating the transmembrane helix, TM, in beige, the N‐domain residing in the mitochondrial matrix domain in red, the AAA+ domain in magenta, and the protease domain in yellow. B) Cryo‐EM structure of the YME1 hexamer (PDB‐ID: 6AZ0) residing in the mitochondrial membrane. C) Schematic representation of the structural elements of the *m*‐AAA monomer indicating the transmembrane helices, TM1 and TM2, in light‐green, the IMS‐domain in light‐blue, the AAA+ domain in purple, and the protease domain in green. D) Cryo‐ EM structure of the AFG3L2 hexamer (PDB‐ID: 6NYY) residing in the mitochondrial membrane.

Initially, the amino‐terminal domain of *S. cerevisiae* (*S. cerevisae)* YME1 has been characterized using solution NMR spectroscopy, employing a truncated part of the amino‐terminal core (residues 97–177) of the protein, which formed a stably folded structure in solution. The structure revealed five α‐helices, with helices 2 and 3 as well as helices 4 and 5 forming TPR (tetratricopeptide repeat) hairpins,^[138]^ which are ubiquitous domains involved in peptide binding.^[178]^ The distinct structure of this domain compared to bacterial FtsH as well as the predicted structures of other homologous proteins shows a large variance of this part of the protein lacking annotated function, despite large structural resemblance of the other modules to the FtsH‐like proteases.^[138]^ Subsequently, the structure of the yeast YME1 carboxy‐terminal domain, bound to nucleotides, was solved by cryo‐EM at 3.4 Å resolution (Figure 5A, B). In this study, the transmembrane spanning region was replaced with a peptide that forms a hexameric coiled coil in solution and thus oligomerizes the AAA+ and the protease domain.[^179^, ^180^] While the protease domain displayed a symmetric ring structure, the AAA+ domain was found to assemble into a spiral staircase.^[180]^ The AAA+ subdomains are progressively rotated and translated with respect to one another, marking potentially a distinction to bacterial FtsH where the AAA+ domain arrange in symmetric hexamer.[^134^, ^135^, ^180^] Despite these differences, it has to be noted that they might be dependent on the nucleotide bound as the available structure of YME1 is in the ATP‐bound state whereas for FtsH only the apo‐ and ADP‐bound state structures are reported thus far.[^134^, ^135^, ^180^]

The structure gave rise to a potential substrate translocation mechanism in which ATP hydrolysis occurs stepwise in a counterclockwise process around the hexamer.[^171^, ^180^] Nucleotide binding occurs at the interface of two neighboring AAA+ domains and in the presence of a substrate, three nucleotide states were observed in the structure. In the spiral staircase, four subunits were found to have ATP bound, the lowest subunit instead bound an ADP, and the “step” subunit displayed an apo‐state with no nucleotides bound.[^171^, ^180^] The AAA+ domain that contains a characteristic pore loop 1 motif ΘXG (Θ is an aromatic residue, X is any residue, G: glycine) has previously been shown to play an important role in substrate unfolding and translocation as it undergoes conformational changes upon ATP hydrolysis.[^144^, ^181^] In YME1, the conserved aromatic residue is a tyrosine (Tyr354) which was shown to follow the pattern of the AAA+ domain, forming a spiral staircase where it directly interacts with the substrate. Furthermore, the conformation of pore loop 1 directly correlates to the nucleotide state of the respective subunit. It was found that the pore loop of ATP bound subunits formed interactions with the substrate. With ATP binding to four subunits, their pore loops engaged with the substrate to transport it through the central pore into the proteolytic chamber.^[180]^

Besides this novel structural information biochemical studies employing a soluble hexameric variant of YME1 could reveal detailed insight into the recognition and processivity of *i*‐AAA proteases.[^179^, ^180^, ^182^] A degron enriched with aromatic residues (FAWFP) could be identified and the existence of partially unfolded state could be derived as a prerequisite for proteolytic cleavage by YME1 .[^179^, ^180^, ^182^, ^183^] Subsequent work employing a similar soluble hexameric human YME1L variant for NMR spectroscopy, dynamic light scattering, and hydrogen‐deuterium mass spectrometry investigations could reveal an allosteric coupling between AAA+ and proteolytic domain modulated by stabilization of the former through the bound nucleotides.^[184]^

### 6.2 Mitochondrial m‐AAA Proteases

A cryo‐EM structure of homohexameric AFG3L2, lacking the transmembrane helices, with a bound substrate at 3.1 Å resolution was reported (Figure 5C, D).^[185]^ Like YME1, the structure revealed a spiral AAA+ domain on top of a symmetric, planar protease domain. Identification of Phe381 as the conserved aromatic residue in pore‐loop 1 and that this residue only forms contacts with the substrate within ATP bound subunits confirms that the translocation mechanism suggested for YME1 also applies for *m*‐AAA proteases.^[185]^ Although the structure and mechanism of YME1 and AFG3L2 show many similarities, the *m*‐AAA pore loop architecture slightly differs to that of the *i*‐AAA.^[185]^ In AFG3L2, the methionine neighboring Phe381 (Met380) forms a connection to the aromatic residue of the adjacent subunit in a counterclockwise manner. These contacts develop a chain of residues which enclose the substrate and modulate the substrate translocation through the central pore.^[185]^ Although this feature has not been observed in the *i*‐AAA YME1, its general importance has been established in FtsH‐type proteases such as the *m*‐AAA homologs YTA10 and YTA12 in yeast.[^185^, ^186^]

In a similar approach used for the *i*‐AAA protease before also the nature of the degradation signals for AFG3L2 could be identified.[^179^, ^187^] This study highlighted the preference for hydrophobic and small polar residues with the lack of clear apparent degron. The study rather highlighted two key aspects underlying AFG3L2 substrate specificity, *(i)* discrimination of substrates based on their accessible sequences and *(ii)* cleavage dependent on the sequence in proximity to the cleavage site.^[187]^

## 7 Presenilin within the γ‐secretase complex

In mammals, presenilin is part of the γ‐secretase complex where it is responsible for the proteolytic function of the complex.^[188]^ Presenilin (PS) belongs to the aspartyl family of IMPRs and constitutes of two isoforms PS1 and PS2, respectively.[^188^, ^189^] Although both variants are present **in** the γ‐secretase complex, they have shown different levels of proteolytic activity *in vivo* and in their subcellular location.[^190^, ^191^] The P1 γ‐secretase complex is localized to the plasma membrane while the P2 complex is present in late endosomes as well as lysosomes.[^190^, ^191^] The PS isoforms have been extensively studied due to the role of the γ‐secretase complex in Alzheimer's disease as it cleaves the amyloid precursor protein (APP) to generate amyloid‐β peptides.[^192^, ^193^] Furthermore, the complex is involved in the cleavage of the Notch receptor, which is implicated in a large variety of cancer types.[^194^, ^195^, ^196^]

The first structural information about presenilins could be derived from the crystal structure of the archaeal homolog PSH1.^[197]^ The structure revealed a large water filled hole that traverses the entire protein across the lipid bilayer, allowing passage of small ions, that is surrounded by helices TM2, TM3, TM5, and TM7.[^197^, ^198^] This structure could corroborate the ongoing debate at the time that PS1 is involved in Ca^2+^ leakage from the ER.[^199^, ^200^] The active site is formed by Asp162 in TM6 and Asp220 in TM7 within the characteristic sequence motifs ΦYDΦΦ in TM6 and ΦGΦGD, where Φ represents hydrophobic residues.[^189^, ^197^] For the human variants, it could be shown that presenilins undergo endoproteolytic self‐cleavage after TM7.[^201^, ^202^]

**Figure 6:**
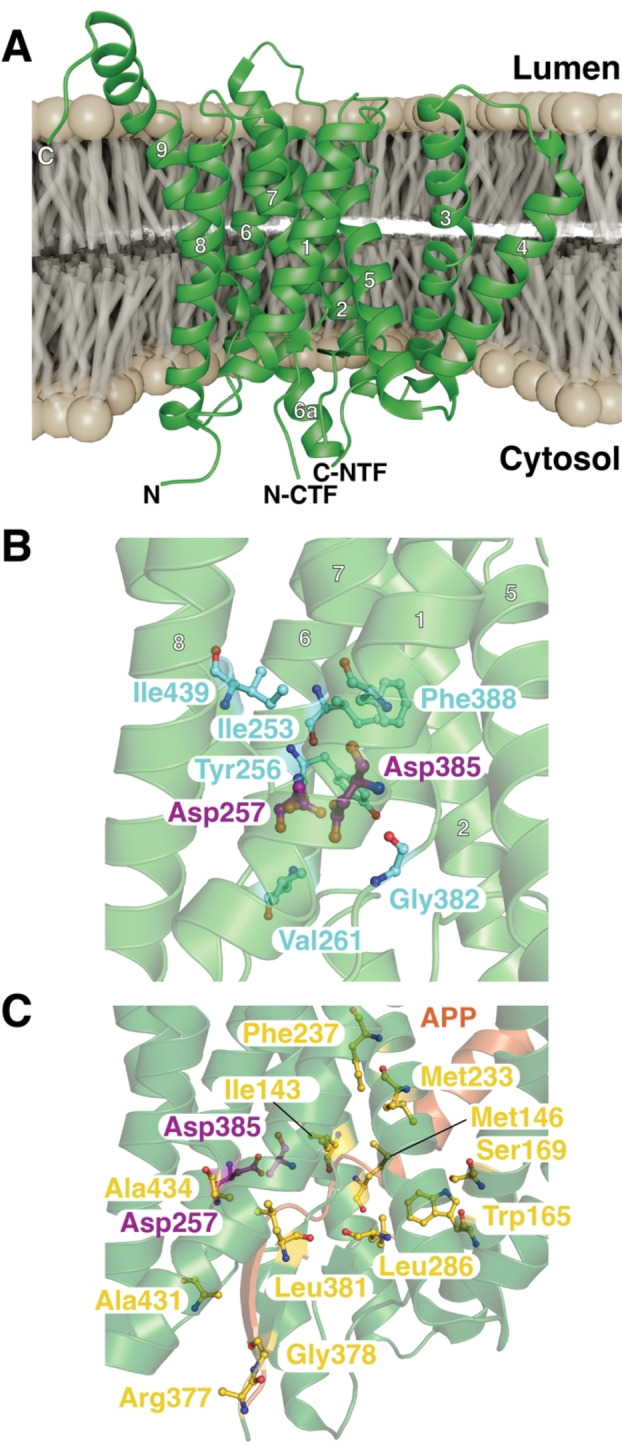
A) Cryo‐EM structure of mature Presenilin1 (PDB‐ID: 7Y5T) represented in the plasma membrane. The individual helices, boundaries of the CTF (carboxy‐terminal fragment) and NTF (amino‐terminal fragment) as well as the termini are marked. B) Zoom in onto the catalytic site residues Asp257 and Asp385 (purple). Highly conserved residues lining up the catalytic cavity are shown in cyan. C) Cryo‐EM structure of Presenilin1 (green) in the presence of APP (amyloid precursor protein; orange) (PDB ID: 6IYC). Frequently mutated residues lining up the substrate binding groove are shown in yellow.

For the resulting carboxy‐terminal fragment (CTF), solution NMR spectroscopy was subsequently used to determine its structure in detergent micelles.^[203]^ The isolated CTF structure indicated unique structural features for PS1: TM7 bearing one of the catalytic aspartic acids only half‐transversing the membrane and a kinked helix TM9.^[203]^ Initial cryo‐EM structures of the γ‐secretase suggested local flexibility in the proximity of the active site, mainly in TM2 and TM6 (Figure 6A, B).[^204^, ^205^] The first complete structures of presenilin were obtained within the γ‐secretase complex, formed from presenilin‐1 together with Nicastrin, Aph‐1 and Pen‐2^[188]^ by cryo‐EM bound to either Notch or APP (amyloid precursor protein) substrates.[^206^, ^207^] Both structures showed an extended conformation stabilized by β‐strands of both presenilin NTF and CTF for the amino‐terminal half of the otherwise α‐helical transmembrane domains of APP, by an extended substrate binding groove lined by Alzheimer's disease related mutations (Figure 6C).^[207]^ Remarkably, the observed presenilin conformation matched an earlier structural study of the protease in the presence of a dipeptide inhibitor.^[208]^ In general, γ‐secretase represents a potential therapeutic target for Alzheimer's disease.^[209]^ Thus, latest structural studies focus on the characterization of different secretase inhibitors such as former clinical candidates semagecestat and avagacestat.^[210]^ The obtained structures indicated that the inhibition was due to blocking a key aspect of the substrate‐enzyme interaction, the β‐strand augmentation observed in the complex structures reported earlier.[^206^, ^207^] In a parallel study focusing on another compound, the presenilin1 selective inhibitor MRK560, revealed sequence dependence in the substrate binding site of presenillin1 as the inhibition depended on Thr281 and Leu282.^[211]^ In parallel, the apoPS2 structure was determined, revealing a virtual identical structure as reported for apoPS1 previously.[^205^, ^211^] The only marked difference is the lack of resolution for the complete TM2 in presenilin2, suggesting extensive inherent flexibility.^[211]^

## 8 Ras Converting Enzyme 1 (Rce1)

Ras converting enzyme 1 (Rce1) represents the newest class of IMPRs as the first crystal structure in 2013 revealed a glutamyl catalytic mechanism that distinguished Rce1 from all other existing integral membrane protease families.^[11]^ In eucaryotes, this protein is localized in the endoplasmic reticulum (ER) and homologs are found in all three domains of life.^[212]^ The protease was initially identified in *S. cerevisiae* where it was found to be involved in the processing of both Ras protein and the mating pheromone **a**‐factor.^[213]^ Nowadays, it is known that Rce1 is involved in a large variety of biological processes, as it is required for the maturation of proteins constituting a carboxy‐terminal CaaX motif (C: cysteine, a: aliphatic residue, X: any carboxy‐terminal residue).^[214]^ Proteins carrying this motif usually undergo a series of post translational modifications (PTMs) initiated by isoprenylation, where either farnesyl‐ or geranylgeranyl groups attach to the cysteine, the second step includes endoproteolysis of the aaX motif and lastly, carboxyl methylation. In this chain of events, Rce1 is responsible for the removal of the carboxy‐terminal aaX residues.[^213^, ^215^] Members of the CaaX protein family include, RAS GTPases, heterotrimeric G proteins, nuclear lamins as well as a number of different phosphatases and kinases.^[216]^ Caax bearing proteins are involved in a broad range of cellular processes such as, inhibition and progression of cancer, ageing, parasitic growth, and cell division.[^217^, ^218^] Homologous proteins, such as the cell wall hydrolase *Sa*SpdC, important for integration of peptidoglycan into the cell wall, and *Sa*MroQ, important for pheromone biosynthesis, are widespread in bacteria but their common function remains to be determined.[^219^, ^220^]

Despite its importance, knowledge about Rce1’s structure remained severely limited. The topology of *S. cerevisiae* Rce1 (*Sc*Rce1) was predicted to consist of seven or eight transmembrane helices with the amino‐terminus facing the ER lumen and the carboxy‐terminus oriented into the cytosol.^[221]^ The study is based on bioinformatic analysis in combination with maleimide cysteine reaction probing, and hypothesizes at least one membrane spanning helix at the amino‐terminus, which is followed by tightly folded cytosolic domains and/or re‐entrant helices separated by loop regions.^[221]^

**Figure 7:**
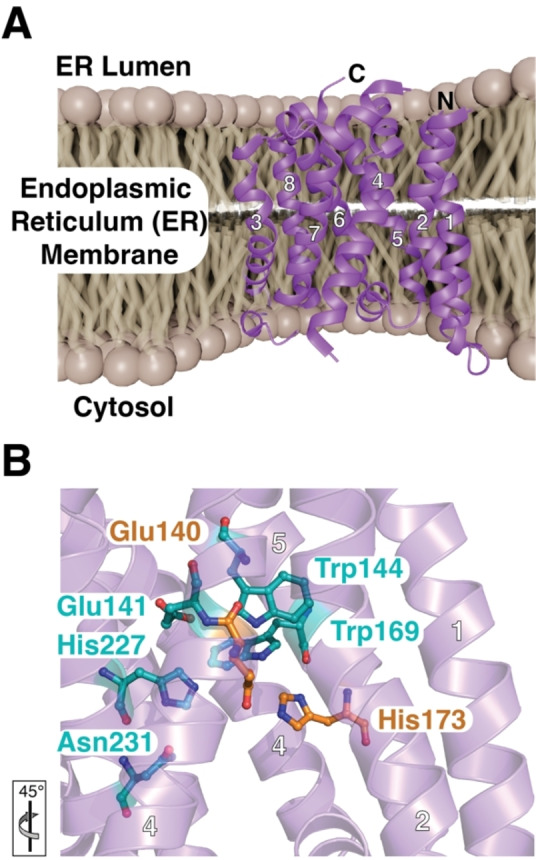
A) Crystal structure of the Ras converting enzyme (Rce1, PDB‐ID: 4CAD) represented in the endoplasmic reticulum membrane. The individual helices and termini are marked. B) Zoom in onto the catalytic site residues Glu140 and His173 (orange). Highly conserved residues lining up the catalytic cavity are shown in cyan. See main text for details. The orientation relative to panel A is indicated.

So far, the only available three‐dimensional structure of an Rce1 protein is from the archaeon *Methanoccocus maripaludis* (*Mm*Rce1) which was solved by X‐ray crystallography in complex with an antibody Fab fragment at 2.5 Å.^[11]^ The crystal structure revealed eight conserved TM helices with both the amino‐ and carboxy‐termini facing the ER lumen (Figure 7A).^[11]^ Short loops link the individual TM segments, except for the segments connecting TM2 with TM3 as well as TM7 with TM8, which instead are connected by peripheral α‐helices. TMs 2–8 form an extended cone shaped cavity, with a volume of ~1300 Å^3^, opening up to the cytosolic site being separated from the ER lumen by interactions with Arg145 positioned at the bottom of the cavity.^[11]^ In addition, one side of the cavity is exposed to the membrane *via* an opening in between TM2 and TM4, proposedly to act as the substrate‐lipid interaction site.^[11]^

The active site of Rce1 is located on top of this cavity residing within the membrane and is constituted of the highly conserved residues Glu140, His173, His227 and Asn231 (Figure 7B). Glu140 and His173 form the catalytic dyad and thus are essential for the proteolytic activity, as mutation of either one of these residues severely impairs proteolytic activity.[^11^, ^222^, ^223^] Furthermore, the residues of the catalytic dyad are positioned opposite to each other on different TMs and hold a water molecule, which is activated for nucleophilic attack of the substrate scissile bond .^[11]^ Glutamate‐activated water is for example also used by zinc‐dependent metalloproteases, and the formation of an oxyanion hole by Glu140 and His173 bears a striking resemblance to the proteolytic mechanism of the rhomboid protease GlpG discussed above.[^11^, ^16^, ^25^, ^26^, ^27^, ^28^] The available structural information reveals that Rce1 displays a unique and novel fold among the IMPRs lacking homology to other known structural domains.[^11^, ^224^]

Functionally, it is known that Rce1 cleaves a large variety of proteins constituting the CaaX motif and this well‐defined substrate recognition motif is relatively unique for IMPR substrates. However, much less is known about the basis of substrate recognition and subsequent steps in the catalytic cycle.^[225]^ A study of *Sc*Rce1 based on mutational analysis revealed that the active site residues Glu156, His194 and His248 are crucial for proteolytic function and that mutation of Glu157 and Asn252 severely reduces catalytic activity.^[223]^ These findings were later supported by the crystal structure of *Mm*Rce1, which showed that Glu140 and His173 (Glu156 and His194 of *Sc*Rce1) are critical for catalysis, mutation of either Glu141, His227 or Asn231 (Glu157, His248 and Asn252 *Sc*Rce1) severely impaired function (Figure 7B).^[11]^ In addition, two conserved aromatic residues, Trp144 and Trp169 in *Mm*Rce1, contact the Glu140 sidechain potentially modulating its p*K*_a_ value.^[11]^ Altogether, Rce1 seems to display a novel catalytic mechanism with resemblances to other proteases such as metalloproteinases which also has a catalytic glutamate driving the proteolysis, and with other IMPs where the oxyanion hole also is formed by a histidine and asparagine.[^11^, ^223^]

Elucidation of the *Mm*Rce1 crystal structure led to a proposed substrate recognition mechanism based on a farnesylated CaaX motif of the RhoA peptide. The substrate peptide inserts itself into the central cavity of Rce1 by adopting a β‐hairpin conformation, where the farnesyl group of the CaaX motif seals the opening to the membrane between the non‐polar residues of TM2 and TM4.^[5]^ The substrate scissile bond would then be positioned in direct proximity to the nucleophilic water molecule, bridged by the catalytic residues His173 and Glu140. Nucleophilic attack on the scissile bond would thus be promoted by protonation of the water molecule by the catalytic dyad, Glu140 and His173.^[5]^ Side chains of the two remaining residues in the active site, His227 and Asn231, are proposed to stabilize the resulting oxyanion intermediate. The final step is thereafter catalyzed by either Glu140 or His173 where protonation of the leaving amino group of the AAX motif completes the proteolysis.^[5]^ Although both the structure and underlying proteolytic mechanism of Rce1 are unique, the currently proposed proteolytic mechanism resembles the ones used by the other IMPRs such as bacterial GlpG.^[11]^

## 9 Summary and Outlook

Recent years saw a wealth of novel structural insight mainly provided by cryo‐EM methods leading to the current level of understanding of membrane protein quality control. In parallel, since its release in 2021 the machine learning method AlphaFold2 (AF2), alongside RoseTTA fold, and the subsequent 2024 upgrade AlphaFold3 (AF3) have revolutionized structural biology.[^98^, ^226^, ^227^] Nowadays, high resolution structural predictions of complete proteomes have become available (e. g.^[228]^), raising the important question of a prolonged necessity of laborious structural biology approaches as outlined in this review.^[229]^

Extensive assessment of these artificial intelligence approaches showed that the obtained predictions can provide excellent starting points for hypothesis driven questions, thus accelerating experimental structure determinations but are yet unable to replace them at the current stage (e. g.^[230]^). One of the limitations of AF2 represented initially the high‐resolution prediction of proteins in the presence of ligands and co‐factors, which was only possible after enhancing the original algorithm and was included finally in AF3 enabling broad usage.[^98^, ^231^, ^232^] However, a recent critical assessment of the existing deep‐learning based methodology for predicting protein‐ligand interactions revealed that the algorithms largely memorised ligand poses from their training data sets, thus limiting their usage for *de novo* drug design at the current stage.^[233]^

Another key question still to be answered is the ability of the current algorithms to provide insight into structural adaptations and enabling access into underlying protein dynamics. Although adaptations to AF2 enabled the algorithm to generate structural ensembles instead of ground‐state structures (e. g.[^234^, ^235^]), in particular for proteins undergoing large structural changes such as metamorphic proteins, existing in partially completely different folds, AF2 s ability to reflect experimental data is currently debated.[^236^, ^237^, ^238^] Despite the fantastic capabilities of the currently available algorithms, for integral membrane proteins these have additional key limitations: *(i)* the training set for the algorithms contains more than 90 % of soluble proteins (PDB; www.rcbs.org); *(ii)* current machine learning approaches are not equipped to predict protein structures directly within the hydrophobic environment of biological membranes.

Therefore, experimental determination of structures of IMPRs by cryo‐EM and X‐ray crystallography will likely be rather enriched by artificial intelligence than be made obsolete in the coming years. Furthermore, considering the recent progress and the contribution of high‐resolution NMR spectroscopy in the last decade for protein quality control in general (e. g. reviewed in[^239^, ^240^, ^241^, ^242^]), alongside the seminal studies presented herein for IMPRs, we foresee extensive usage of these techniques to resolve their functional repertoire in the near future. In particular, as NMR spectroscopy is the only experimental technique capable of providing insight at the atomic level into the underlying dynamics of IMPR function over a large range time scales, ranging from picoseconds to real‐time, we are convinced it will enable unprecedented mechanistic insight (reviewed in[^239^, ^243^, ^244^, ^245^]).

In summary, we expect that these future advanced studies will be integrating a large variety of complimentary experimental and computational approaches (reviewed in^[246]^), that will be further facilitated by the availability and more broad usage of more native‐like membrane mimetics, such as different types of nanodiscs as described above and the potential of native membranes as indicated by initial pioneering studies.^[247]^

## Conflict of Interests

The authors declare no conflict of interest.

## Biographical Information

*Hannah Fremlén obtained her BSc in chemical biology and MSc in protein science and technology from Linköping University, Sweden. She is currently pursuing a Ph.D. in biophysics at Gothenburg University, Sweden, studying the structural and functional details of a bacterial integral membrane protease using nuclear magnetic resonance (NMR) spectroscopy methods. Her research aims to provide structural and dynamical insight into membrane proteins involved in the protein quality control mechanism*.

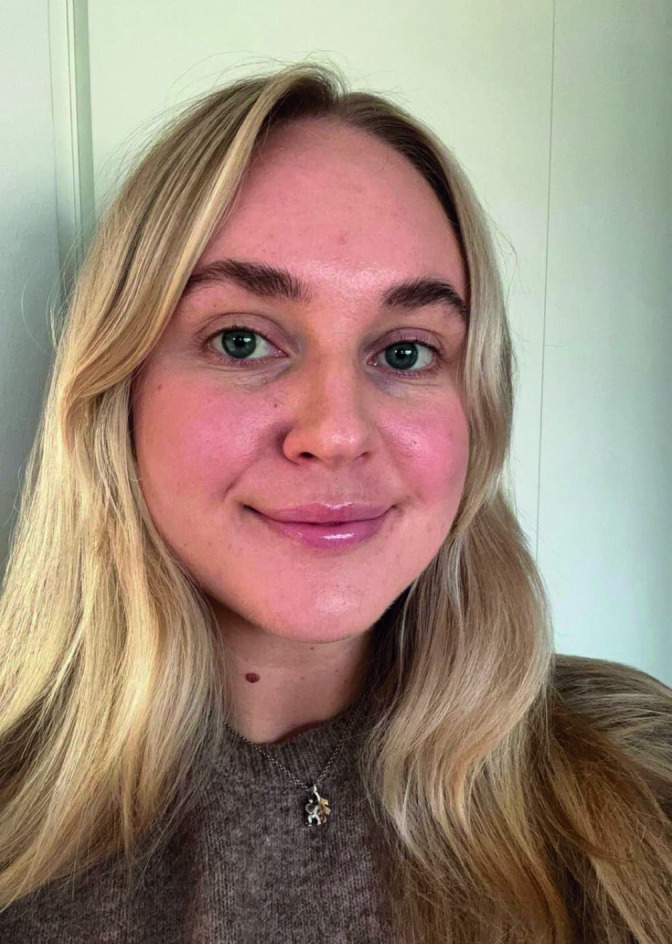

## Biographical Information

*Björn M. Burmann received his Ph.D. in 2010 from the University of Bayreuth, Germany, supervised by Prof. Dr. Paul Rösch, in biophysics. After a postdoctoral period at the Biozentrum Basel, Switzerland, with Prof. Sebastian Hiller, he was in 2017 initially appointed as an Assistant Professor and now is a full Professor at the University of Gothenburg, Sweden. His current research focusses on large molecular machineries involved in the cellular protein quality control. He was named EMBO Young Investigator in 2020 as well as awarded the Anatole Abragam Prize in 2017 and the Sven och Ebba‐Christina Foundation Prize in 2023 for his research*.